# Muscle Strength and Efficiency of Muscle Activities Recovery Using Single-Joint Type Hybrid Assistive Limb in Knee Rehabilitation after Anterior Cruciate Ligament Reconstruction

**DOI:** 10.3390/jcm12196117

**Published:** 2023-09-22

**Authors:** Yuichiro Soma, Hirotaka Mutsuzaki, Tomokazu Yoshioka, Shigeki Kubota, Koichi Iwai, Yukiyo Shimizu, Akihiro Kanamori, Masashi Yamazaki

**Affiliations:** 1 Department of Orthopaedic Surgery, Institute of Medicine, University of Tsukuba, Tsukuba 305-8576, Japan; ysoma@md.tsukuba.ac.jp (Y.S.); tymd99@tsukuba-seikei.jp (T.Y.); kanamori@md.tsukuba.ac.jp (A.K.); masashiy@md.tsukuba.ac.jp (M.Y.); 2 Division of Regenerative Medicine for Musculoskeletal System, Institute of Medicine, University of Tsukuba, Tsukuba 305-8576, Japan; 3 Center for Medical Sciences, Ibaraki Prefectural University of Health Sciences, Ibaraki 300-0394, Japan; 4 Department of Orthopaedic Surgery, Ibaraki Prefectural University of Health Sciences Hospital, Ibaraki 300-0331, Japan; 5 Department of Occupational Therapy, School of Health Sciences, Ibaraki Prefectural University of Health Sciences, Ibaraki 300-0394, Japan; skubota@tsukuba-seikei.jp; 6 Faculty of Health and Medical Sciences, Ibaraki Prefectural University of Health Sciences, Ibaraki 300-0394, Japan; iwai@ipu.ac.jp; 7 Department of Rehabilitation Medicine, Institute of Medicine, University of Tsukuba, Tsukuba 305-8576, Japan; shimiyukig@md.tsukuba.ac.jp

**Keywords:** ACL reconstruction, knee HAL single-joint training, isokinetic muscle strength

## Abstract

Decreased muscle strength often occurs after anterior cruciate ligament (ACL) reconstruction; this can include muscle atrophy, neuromuscular dysfunction, and reduced force generation efficiency. Hybrid assistive limb (HAL) technology, which integrates an interactive biofeedback system connecting the musculoskeletal system to the brain and spinal motor nerves, offers a potential intervention. Our study, conducted from March 2018 to August 2023 using knee HAL single-joint technology, was a prospective non-randomized controlled trial involving 27 patients who had undergone arthroscopic ACL reconstruction. They were split into two groups: HAL (18 patients) and control (nine patients). Beginning 18 weeks after their surgery, the HAL group participated in three weekly sessions of knee HAL-assisted exercises. Both the HAL and control groups underwent isokinetic muscle strength tests at postoperative weeks 17 and 21. Testing utilized an isokinetic dynamometer at 60°/s, 180°/s, and 300°/s. The Limb Symmetry Index (LSI) was employed to measure side-to-side differences. The HAL group showed significant LSI improvements in peak extension torque across all testing velocities and for peak flexion torque at 60°/s and 300°/s. The rate of change in LSI for peak flexion torque at 300°/s was significantly higher post-measurements (*p* = 0.036; effect size = 1.089). The change rate for LSI in peak extension torque at 300°/s and all peak flexion torques showed medium to large effect sizes in Cohen’s d. In conclusion, knee HAL single-joint training positively influenced muscle strength recovery and efficiency. The HAL training group exhibited superior muscle strength at various isokinetic testing velocities compared to the control group.

## 1. Introduction

Anterior cruciate ligament (ACL) injury is the most common among sports injuries. ACL reconstruction is performed on patients with this injury to restore knee joint stability, improve self-reported function, and facilitate a safe return to sports [1]. Decreased muscle strength often occurs after ACL reconstruction. This decrease can be attributed to muscle mass atrophy and reduced efficiency in muscle force generation due to factors like neuromuscular dysfunction and muscle stiffness [2]. Biochemical investigations have revealed alterations in the tensile strength of series elastic components subsequent to disuse caused by orthopedic surgery in human subjects [3,4]. Concurrently, additional research has documented a decrease in muscle fiber stiffness after a period of no weight bearing in rodent models. Diminished stiffness within the series elastic components has the potential to induce a decline in their efficacy of transmitting contractile tension to bones. Consequently, this could contribute to a diminished muscular strength during physical activities [5]. Moreover, the ACL contains mechanoreceptors that influence muscle strength, proprioception, and recruitment patterns [6,7]. Deficiency of the ACL may lead to various neuromuscular issues, one of which is arthrogenic muscle inhibition, a condition characterized by the failure of quadriceps activation due to neural dysfunction [8].

The HAL single joint is an exoskeleton-type wearable robot that provides voluntary assistive training. It uses an actuator on the lateral side of the knee joint and detects muscle action potentials from the middle fibers of the quadriceps and hamstrings. This technology incorporates an interactive biofeedback system, linking the musculoskeletal system with motor nerves associated with the brain and spinal cord [9]. Previous research on HAL single-joint training suggests that sensory feedback, resulting from physiologically accurate motion, aligned with physiological predictions, can prompt modifications in the central nervous system. These changes might adjust muscle recruitment patterns, aiding in functional recovery [10]. Our previous research on knee HAL single-joint training for patients post-ACL reconstruction indicated that this training method may potentially be a safe rehabilitation tool, enhancing muscle activity and the efficiency of muscle activities [11]. Based on these findings, we hypothesized that knee HAL training might lead to improved peak muscle torque by coordinating or decreasing a high level of muscle co-contractions and balancing the activities of hamstring and quadriceps muscles, as observed in the electromyography data from ACL-reconstructed knees [11]. However, it is challenging to distinguish the recovery due to ACL reconstruction from the effect of knee HAL single-joint training. Therefore, including a control group is crucial to assess these effects accurately. We hypothesized that knee HAL single-joint training may optimize muscle activities, resulting in muscle strength differences compared to the control group at different isokinetic muscle strength testing velocities. In this study, we aimed to investigate the differences in the physical assessments, including isokinetic muscle strength, between the HAL and control groups in patients with ACL reconstruction.

## 2. Materials and Methods

### 2.1. Patients

We conducted a prospective non-randomized controlled trial at our institution between March 2018 and August 2023. During this time, all patients who had undergone arthroscopic ACL reconstruction were assessed for eligibility.

The inclusion criteria of the HAL group were a primary ACL injury; the ability to comprehend the study’s explanation and provide informed consent; and consistent availability for the study’s duration. Patients with multiple knee ligament injuries, those who might find it difficult to wear and train with the HAL due to underlying diseases, and those who experienced perioperative complications were excluded. The HAL group consisted of 18 patients (ten men, eight women; mean age, 23.4 ± 7.0 years; height, 168.0 ± 8.9 cm; weight, 66.7 ± 13.0 kg). The inclusion criteria of the control group were a primary ACL injury; the ability to comprehend the study’s explanation and provide informed consent; and consistent availability for the study’s duration. Patients with multiple knee ligament injuries and those who experienced perioperative complications were excluded. These patients had undergone arthroscopic ACL reconstruction using soft tissue graft materials, including anatomic single bundle (*n* = 16) and anatomic double bundle (*n* = 2). The control group comprised 9 patients (three men, three women; mean age, 20.2 ± 1.7 years; height, 162.7 ± 10.0 cm; weight, 64.9 ± 10.7 kg). All of them had undergone arthroscopic ACL reconstruction with the anatomic single bundle. In both groups, patients had undergone arthroscopic ACL reconstruction under the supervision of the same orthopedic surgeon within our institution. The multi-stranded grafts were obtained either from the semitendinosus tendon alone or in combination with the gracilis tendon. The tendon graft was attached to the Ultrabutton adjustable-loop device (Smith & Nephew Endoscopy, Andover, MA, USA) on the femoral side, while the tibial end was sutured using a double-spike plate and screw (Smith & Nephew Endoscopy). This was performed with an initial tension of 20 N or 30 N, using a tension meter set at 20° or 30° of knee flexion. The bone tunnels on the femoral and tibial sides were created anatomically at the ACL’s insertion points using the outside-in tunnel technique. All patients started their rehabilitation training with a physical therapist on postoperative day 1. From postoperative day 5, they began range of motion (ROM) and weight-bearing exercises. After discharge, the rehabilitation program was administered once a week by either a physical therapist or an athletic trainer. This program included closed and open kinetic chain exercises, strength training for the hip and knee muscles, neuromuscular training, neuromuscular electrical stimulation, and cryotherapy for the ACL-reconstructed leg. After a 3-month follow-up, patients in competitive sports continued with the rehabilitation program two or three times a month, while those in recreational sports received it once a month. The frequency and content of inpatient and outpatient rehabilitation following ACL reconstruction were analogous in both groups. We chose not to control the rehabilitation program to maintain the external validity of our findings. Table 1 summarizes the patients’ clinical characteristics.

### 2.2. Knee HAL Single-Joint Training

The knee HAL single-joint training began 18 weeks post-ACL reconstruction to avoid the risks associated with graft tension laxity, partial graft tears, and insufficient synovial coverage [12,13]. The knee HAL single-joint training was performed once a week for a total of three sessions. Prior to setting up, the physical therapist took measurements of the patient’s femoral and lower limb lengths, widths of the hip and ankle, and the maximum active flexion and extension ROM of the reconstructed ACL leg. While the patient was seated, the therapist fitted each leg attachment and ankle support (fitting time, 3–5 min). After measuring the maximum active flexion and extension angles, the knee HAL assist angle was adjusted to prevent over-assistance prior to intervention. For extension, the HAL assist angle was adjusted to −5 degrees from the maximal active extension angle. In contrast, the HAL assist flexion angle was set to 120 degrees for all patients, corresponding to the maximum flexion angle. Surface electrodes were attached to the quadriceps and hamstring muscles (vastus medialis, rectus femoris, vastus lateralis, and biceps femoris, semitendinosus) to evaluate the bioelectrical activity from the long axis (along the belly) of each muscle. The electromyogram waveform was observed during knee joint flexion and extension with HAL to confirm muscle contractions. Before the first knee HAL training session, patients were instructed to perform smooth and comfortable leg extension and flexion movements. Subsequently, the assistance level for the knee HAL single-joint training was determined [14]. During the knee extension training, patients were seated at the end of a bed, and for flexion training, they were in a prone position. They underwent five sets of HAL-assisted knee extension and flexion exercises (10 exercises/set, totaling 50) [11]. Each session lasted approximately 50 min including fitting and evaluation. Conventional rehabilitation was provided on days without HAL training.

### 2.3. Measurements

**Figure 1:**
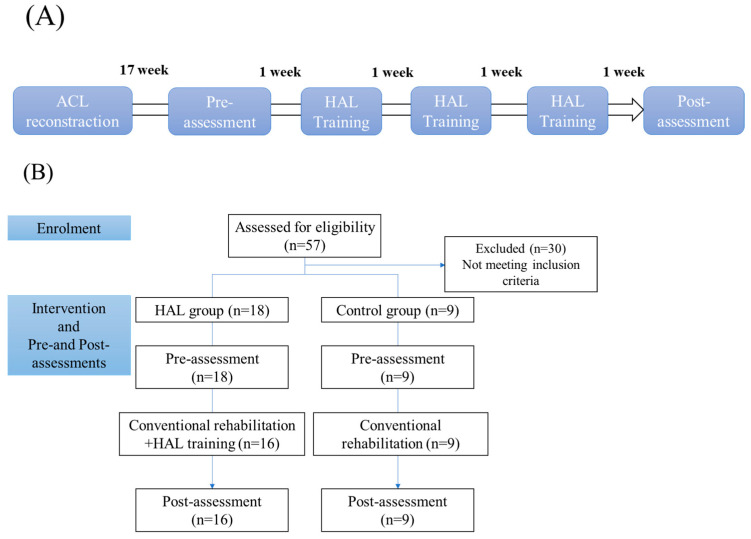
(**A**) Postoperative progress and duration of the knee hybrid assistive limb single-joint training following anterior cruciate ligament reconstruction. (**B**) Flow of participants through the intervention.

Both the HAL and control groups underwent isokinetic muscle strength assessments during postoperative weeks 17 and 21 (Figure 1A,B). The strength assessments were performed at 60°/s, 180°/s, and 300°/s using an isokinetic dynamometer (Biodex System III; Biodex Medical Systems, Sakai Inc., Tokyo, Japan). Strength measurements were obtained during both knee extension and flexion, bilaterally. The Limb Symmetry Index (LSI) was calculated to determine side-to-side differences. The LSI represents the ratio of the injured side to the non-injured side and is expressed as a percentage (injured/non-injured × 100%). This value can be classified as either normal or abnormal [15]. In both groups, measurements were taken for active and passive ROM, which were measured using a goniometer; the Tegner Activity Scale score (a numerical grading system for work and sport activities) [16]; the Lysholm Knee Questionnaire score (a 100-point scoring system to assess knee-specific symptoms) [16]; and the International Knee Documentation Committee subjective knee form score (a scoring system to quantify disability caused by ACL injury) [17].

### 2.4. Statistical Analysis

The independent-samples *t*-test and Fisher’s exact test were utilized to evaluate the patients’ clinical characteristics between the HAL and control groups for each measurement. In the HAL and control groups, the paired-samples *t*-test was utilized to evaluate LSI differences between the pre- and post-assessment and the differences in the physical assessments between the pre- and post-assessment. The independent-samples *t*-test was utilized to evaluate the pre- and post-assessment of LSI and hamstring/quadriceps ratio, the rate of LSI change from pre to post, and to evaluate the differences in the physical assessments between the HAL and control groups. The LSI’s rate of change was calculated and subjected to the *t*-test. Independent *t*-tests determined muscle strength result differences between the HAL and control groups for each measurement (pre-assessment, post-assessment, and the pre-post rate of change of the LSI). Additionally, a two-way analysis of variance compared the LSI results between the pre- and post-assessments of both the HAL and control groups. The LSI, LSI’s rate of change, and hamstring/quadriceps ratio were calculated with a power analysis using the G*Power software (latest ver. 3.1.9.7). In addition, the LSI, ROM, Tegner Activity Scale, and Lysholm Knee Questionnaire scores were calculated as effect sizes in Cohen’s d. The statistical analysis was performed using IBM SPSS Statistics 24 software (IBM Corp., Armonk, NY, USA). The alpha level was set at 5%.

### 2.5. Sample Size

This research constitutes an exploratory investigation. As a result, we conducted a sample size calculation utilizing G*Power software (latest version 3.1.9.7). This calculation was based on parameters derived from the rate of change in the LSI for peak flexion torque at 300°/s, including mean values (mean1: 15.07, mean2: −7.21) and a standard deviation (σ) of 14.79. The effect size was determined and the power set at 0.8, with an allocation ratio of 2:1, all to estimate the required sample size. The minimum sample size required to achieve a power exceeding 0.8 in the primary analysis was projected to be 13 and 7 for the respective groups. To account for potential participant attrition, we established a target sample size of 18 and 9 participants, respectively.

## 3. Results

Between March 2018 and August 2023, 18 participants were assessed for eligibility in the HAL group (Figure 1B). At the start of the intervention, two participants withdrew from the study. One had sustained a moderate lateral knee injury, while the other contracted COVID-19. The results of applying the independent-samples *t*-test and Fisher’s exact test to evaluate the patients’ clinical characteristics between the HAL and control groups for each measurement are presented in Table 1. No significant deference in the patients’ clinical characteristics was detected between the HAL and control groups.

**Figure 2:**
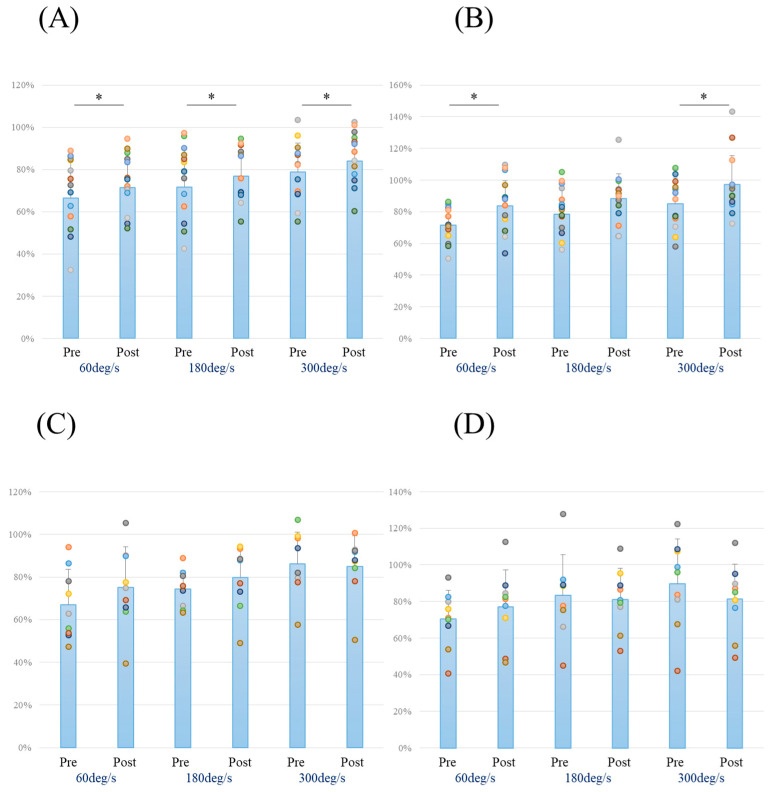
Limb Symmetry Index (LSI) for the HAL group at (**A**) peak extension torque and (**B**) peak flexion torque at each velocity. LSI for the control group at (**C**) peak extension torque and (**D**) peak flexion torque at each velocity. Abbreviations: HAL = hybrid assistive limb; Pre, pre-assessment; Post, post-assessment. * Significant difference (*p* < 0.05).

**Figure 3:**
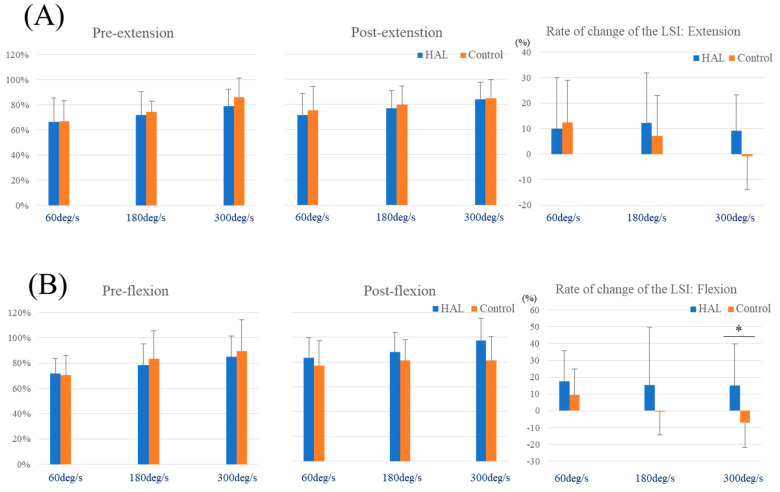
Independent *t*-tests comparing muscle strength between the HAL and control groups at each stage (pre-assessment, post-assessment, and the rate of LSI change from pre to post). (**A**) Peak extension torque of the LSI and the rate of change of the LSI for peak extension torque and (**B**) peak flexion torque of the LSI and the rate of change of the LSI for peak flexion torque at each velocity. HAL, hybrid assistive limb; LSI, Limb Symmetry Index. * Significant difference (*p* < 0.05).

**Figure 4:**
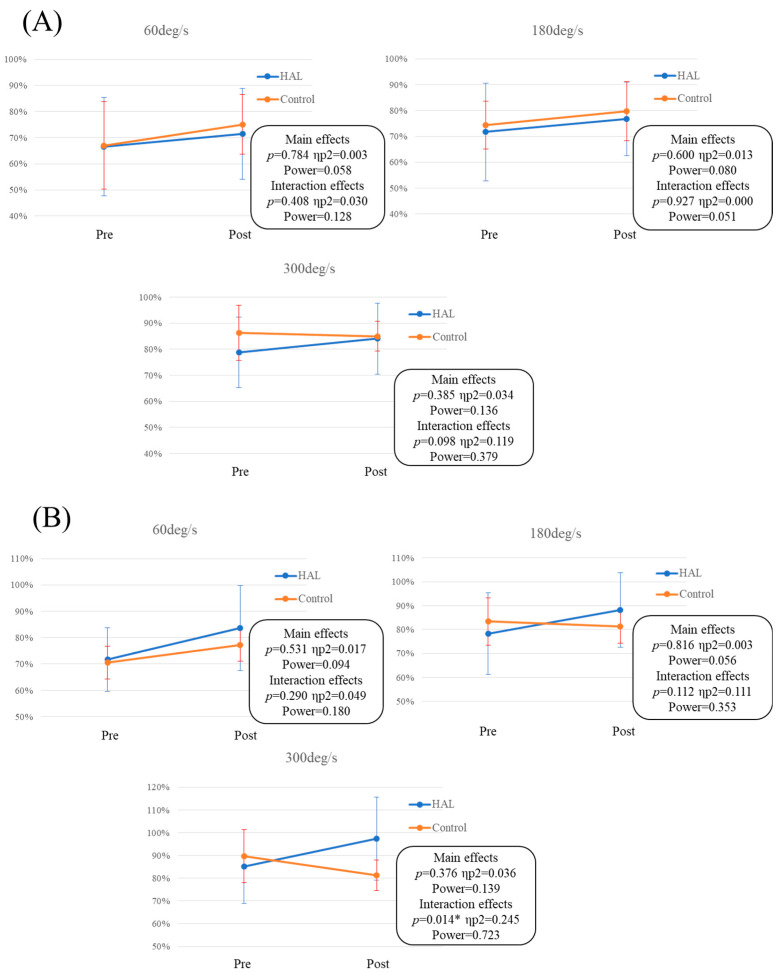
Two-way analysis of variance of LSI between pre- and post-assessments for both HAL and control groups. (**A**) Peak extension torque and (**B**) peak flexion torque at each velocity. HAL, hybrid assistive limb; Pre, pre-assessment; Post, post-assessment. * Significant difference (*p* < 0.05).

In the HAL group, the LSI was significantly higher post-assessment for peak extension torque across all velocities (60°/s; *p* = 0.024, 180°/s; *p* = 0.037, 300°/s; *p* = 0.018) (Figure 2A) and for peak flexion torque at 60°/s (*p* = 0.001) and 300°/s (*p* = 0.049) (Figure 2B). On the other hand, in the control group, the LSI was not significantly higher post-assessment for peak extension and flexion torque across all velocities (Figure 2C,D). Independent *t*-tests comparing isokinetic muscle strength between the HAL and control groups at each velocity are detailed in Table 2 and depicted in Figure 3. The independent *t*-tests revealed a substantial effect size for the post-peak flexion torque especially at 300°/s (*p =* 0.052; *d* = 0.865). Additionally, the rate of change of the LSI for peak extension torque at 300°/s and all peak flexion torque displayed either a medium or large effect size according to Cohen’s d (extension at 300°/s; *d* = 0.728, flexion at 60°/s; *d* = 0.464, 180°/s; *d* = 0.552). Specifically, the rate of change of the LSI for peak flexion torque at 300°/s showed significant differences (*p =* 0.023; *d* = 1.031) (Table 2, Figure 3B), and the hamstring/quadriceps ratio at 300°/s was significantly different in post-measurements (*p* = 0.043; *d* = 0.905). A two-way analysis of variance for the peak flexion torque at 300°/s revealed significant differences (interaction effects *p =* 0.014, ηp2 = 0.245, power = 0.723) (Figure 4B). The outcomes of the physical evaluations can be found in Table 3. In the HAL group, the active ROM was significantly increased in both extension and flexion; ROM in extension (*p =* 0.006; *d* = 1.644), ROM in flexion (*p =* 0.000; *d* = 1.288). ROM in passive flexion was significantly increased in both extension and flexion; ROM in extension (*p =* 0.000; *d* = 1.633), ROM in flexion (*p =* 0.011; *d* = 0.761). The Tegner Activity Scale and Lysholm Knee Questionnaire scores were significantly increased: Tegner Activity Scale score (*p =* 0.001; *d* = 1.097), Lysholm Knee Questionnaire score (*p =* 0.003; *d* = 0.918). In the control group, the Lysholm Knee Questionnaire score was significantly increased (*p =* 0.011; *d* = 1.361).

## 4. Discussion

In this study, we compared the HAL and control groups post-ACL reconstruction in terms of physical assessments, including isokinetic muscle strength. The HAL group demonstrated a significantly elevated LSI post-HAL in both peak extension torque across all velocities and peak flexion torque at 60°/s and 300°/s. Furthermore, we found that knee HAL single-joint training significantly improved outcomes in the physical evaluations, suggesting its potential as a safe and effective rehabilitative technique to enhance muscle strength recovery. However, differentiating between the recovery due to ACL reconstruction and the effect of the knee HAL single-joint training is essential. Notably, the independent *t*-tests comparing isokinetic muscle strength between the HAL and control groups at all velocities revealed a substantial effect size for the post-peak flexion torque, especially at 300°/s. Similarly, the two-way analysis of variance demonstrated a significant interaction effect for the peak flexion torque at 300°/s. Additionally, a substantial effect size was observed for the rate of change of the LSI in peak extension torque at 300°/s and flexion torque across all velocities. The addition of paired-samples *t*-test results for the LSI in the control group, where differences were insignificant, and in the HAL group, where they were significant except at 180°/s, provides valuable context. These insights underscore the impact of HAL intervention within the analysis according to group. A previous study suggested that nerve endings in the reconstructed ligament recover after a minimum of 18 months post-ACL reconstruction, which was demonstrated by the detection of somatosensory evoked potentials upon stimulating the reconstructed ACL and observed improvement in knee joint position sense on the injured side [18,19]. The single-limb hop test provides objective information regarding neuromuscular deficits post-ACL reconstruction [20,21]. Another study correlated isokinetic flexion strength deficits at 180°/s and 300°/s, as well as flexion-to-extension ratios, with hop test performance post-ACL reconstruction [22]. Our findings suggest that other factors, like graft materials, in addition to balance and proprioception, can influence isokinetic extension and flexion asymmetries. Generally, post-ACL reconstruction can lead to multiple anatomical challenges, such as graft tension laxity, partial graft tears, insufficient synovial coverage, and cyclops-like lesions [23]. The transformation of semitendinosus tendon autografts into a histologic structure usually takes at least 12 weeks post-surgery [13]. HAL utilizes a technology that combines voluntary drive and normalized motion assistance from an external device, creating a foundation for a proprioceptive feedback loop [24,25]. This is particularly beneficial for patients with spinal reflex pathway lesions involving reciprocal inhibition or γ-motor and α motor neurons. Neural activity combined with repetitive task execution promotes and potentially restores or restructures appropriate proprioceptive feedback learning [26]. Therefore, in this study, despite starting the protocol 18 weeks post-ACL reconstruction, knee HAL single-joint training exhibited potential benefits in muscle strength recovery and activities efficiency. This impact could be achieved through improvements in neuromuscular coordination, a decrease in muscle stiffness, and addressing deficits in spinal reflex pathways [2,8,27]. This study had several limitations that should be acknowledged. First, the sample size was relatively small, necessitating further research to validate the generalizability of the findings. However, we calculated the effect size using Cohen’s d and assessed the statistical power, which indicated moderate to large effect sizes, confirming that the study had sufficient power given the sample size. Second, our research mainly concentrated on physical assessments and isokinetic muscle strength, not including electrophysiological exams such as ultrasound imaging [28] or surface electromyography [29]. These neurophysiological perspectives are important for a more comprehensive understanding of the effects and mechanisms involved. Future research should consider incorporating these measures to provide a more robust analysis of the outcomes. The duration of the follow-up period in this study is comparatively brief. Considering this limitation, further research endeavors should encompass extended follow-up intervals, spanning up to 1- or 2-years post-ACL reconstruction. Such investigations could encompass variables like muscle strength or even the incidence rate of subsequent ACL injuries.

## 5. Conclusions

Knee HAL single-joint training could potentially optimize muscle activities, resulting in muscle strength differences compared to the control group at each velocity in isokinetic muscle strength tests. Even when initiated 18 weeks after ACL reconstruction, knee HAL single-joint training still offers a positive impact on the recovery of muscle strength and efficiency of muscle activities.

## Figures and Tables

**Table 1:** Patients’ clinical characteristics.

	HAL Group	Control Group	*p*-Value
Age (year)	23.4 ± 7.0	20.2 ± 1.7	0.202
Sex (male/female)	10/8	3/6	0.411
Height (cm)	168.0 ± 8.9	162.7 ± 10.0	0.205
Weight (kg)	66.7 ± 13.0	64.9 ± 10.7	0.588
BMI (kg/m^2^)	23.5 ± 3.5	24.6 ± 4.2	0.655
Injured side (right/left)	6/12	4/5	0.671
Graft materials (Single bundle/double bundle)	16/2	9/0	0.520
Sports level (Competitive/Recreational)	9/9	8/1	0.088

HAL, hybrid assistive limb; BMI, body mass index.

**Table 2:** Independent *t*-tests in isokinetic muscle strength testing between the HAL and control groups at each velocity.

		HAL Group		Control Group		*p*-Value	Effect Size (d)	Power
The LSI (%)		Mean	SD	Mean	SD			
Pre-extension	60 deg/s	66.6	18.8	67.1	16.5	0.951	0.026	0.050
	180 deg/s	71.7	18.9	74.4	8.6	0.635	0.166	0.066
	300 deg/s	78.8	13.6	86.3	14.9	0.215	0.532	0.232
Pre-flexion	60 deg/s	71.7	12.0	70.4	15.7	0.638	0.092	0.055
	180 deg/s	78.3	17.1	83.3	22.4	0.479	0.263	0.093
	300 deg/s	85.2	16.2	89.8	24.3	0.155	0.237	0.085
Post-extension	60 deg/s	71.5	17.4	75.1	19.0	0.638	0.199	0.074
	180 deg/s	76.7	14.2	79.8	14.9	0.621	0.212	0.078
	300 deg/s	84.1	13.6	85.0	14.4	0.871	0.069	0.053
Post-flexion	60 deg/s	83.6	16.2	77.1	20.2	0.388	0.367	0.135
	180 deg/s	88.2	15.7	81.2	16.9	0.315	0.433	0.169
	300 deg/s	97.3	18.2	81.3	19.2	0.052	0.865	0.512
The rate of change of the LSI (%)								
Extension	60 deg/s	9.98	19.96	12.41	16.50	0.759	0.129	0.060
	180 deg/s	12.15	19.59	7.08	15.98	0.519	0.276	0.097
	300 deg/s	9.16	14.06	−0.83	13.06	0.098	0.728	0.388
Flexion	60 deg/s	17.58	18.29	9.53	15.43	0.277	0.464	0.187
	180 deg/s	15.44	34.32	−0.39	14.12	0.204	0.552	0.246
	300 deg/s	15.07	24.68	−7.21	14.79	0.023 *	1.031	0.659
Hamstring/quadriceps ratio (%)								
Pre-assessment	60 deg/s	57.8	14.5	52.7	11.6	0.383	0.370	0.136
	180 deg/s	61.2	11.2	62.5	15.1	0.812	0.100	0.056
	300 deg/s	72.4	11.8	68.8	17.1	0.244	0.499	0.209
Post-assessment	60 deg/s	58.6	21.8	52.9	11.4	0.478	0.301	0.107
	180 deg/s	64.5	14.9	58.9	13.2	0.358	0.396	0.149
	300 deg/s	74.0	13.1	60.5	17.3	0.043 *	0.905	0.548

HAL, hybrid assistive limb; LSI, Limb Symmetry Index; SD, standard deviation. * Significant difference (*p* < 0.05).

**Table 3:** Physical evaluation results of pre- and post-assessments.

		HAL Group		*p*-Value	Effect Size (d)	Control Group		*p*-Value	Effect Size
		Pre	Post			Pre	Post		
Active ROM (°)	Extension	−5.3 ± 3.5	−1.9 ± 2.8	0.006 *	1.644	−6.3 ± 2.6	−5.3 ± 2.0	0.234	0.500
	Flexion	129.3 ± 5.2	133.7 ± 4.5	0.000 *	1.288	130.3 ± 12.5	132.0 ± 5.3	0.613	0.202
Passive ROM (°)	Extension	−2.1 ± 2.6	−0.3 ± 1.1	0.000 *	1.633	−1.7 ± 2.0	−1.5 ± 2.1	0.793	0.113
	Flexion	134.7 ± 4.5	139.6 ± 4.8	0.011 *	0.761	135.8 ± 12.1	138.3 ± 6.4	0.361	0.410
Pivot shift test result		-	-			-	-		
Lachman’s test result		-	-			-	-		
Tegner activity scale score		5.0 ± 0.8	5.7 ± 0.7	0.001 *	1.097	5.1 ± 0.7	6.0 ± 1.4	0.111	0.705
Lysholm knee questionnaire score		68.9 ± 6.6	82.5 ± 10.4	0.003 *	0.918	73.1 ± 10.7	83.1 ± 8.7	0.011 *	1.361
IKDC subjective knee form score		A	A			A	A		

HAL, hybrid assistive limb; IKDC, International Knee Documentation Committee; Post, post-assessment; Pre, pre-assessment; ROM, range of motion. * Significant difference (*p* < 0.05).

## Data Availability

The datasets generated and/or analyzed during the current study are available from the corresponding author upon reasonable request.
